# Overproduction of docosahexaenoic acid in *Schizochytrium* sp. through genetic engineering of oxidative stress defense pathways

**DOI:** 10.1186/s13068-021-01918-w

**Published:** 2021-03-16

**Authors:** Xiao Han, Zhaohui Li, Ying Wen, Zhi Chen

**Affiliations:** State Key Laboratory of Agrobiotechnology, College of Biological Sciences, China Agricultural University, Beijing, 100193 China

**Keywords:** *Schizochytrium* sp., Docosahexaenoic acid, Genetic engineering, Oxidative stress defense pathway

## Abstract

**Background:**

Oxidation and peroxidation of lipids in microorganisms result in increased levels of intracellular reactive oxygen species (ROS) and reactive aldehydes, and consequent reduction of cell growth and lipid accumulation.

**Results:**

To reduce oxygen-mediated cell damage and increase lipid and docosahexaenoic acid (DHA) production in *Schizochytrium* sp., we strengthened the oxidative stress defense pathways. Overexpression of the enzymes thioredoxin reductase (TRXR), aldehyde dehydrogenase (ALDH), glutathione peroxidase (GPO), and glucose-6-phosphate dehydrogenase (ZWF) strongly promoted cell growth, lipid yield, and DHA production. Coexpression of ZWF, ALDH, GPO, and TRXR enhanced ROS-scavenging ability. Highest values of dry cell weight, lipid yield, and DHA production (50.5 g/L, 33.1 g/L, and 13.3 g/L, respectively) were attained in engineered strain OaldH-gpo-trxR by shake flask fed-batch culture; these were increases of 18.5%, 80.9%, and 114.5% relative to WT values.

**Conclusions:**

Our findings demonstrate that engineering of oxidative stress defense pathways is an effective strategy for promoting cell robustness, lipid yield, and DHA production in *Schizochytrium*.

**Supplementary Information:**

The online version contains supplementary material available at 10.1186/s13068-021-01918-w.

## Background

Docosahexaenoic acid (DHA) has received increasing research attention during the past two decades because of its beneficial effects on human health. DHA has been shown to reduce cardiovascular disease risk, lower blood pressure, and exert various anti-inflammatory effects [1, 2]. The unicellular marine eukaryote *Schizochytrium* (class Labyrinthulomycetes; family Thraustochytriaceae) is an algae-like microorganism utilized commercially for production of DHA-rich oil and (in dried form) as a source of DHA in animal feeds, human foods, and nutritional supplements [3–5]. Total fatty acids (TFAs) comprise up to 70% of cell weight of *Schizochytrium* sp., and 25–45% of TFAs are DHA [5, 6]. Traditional culture methods and genetic engineering techniques have been used by many research groups to increase DHA production by *Schizochytrium* sp. [7–12].

Oxygen supply plays a key role in cell proliferation and lipid accumulation of *Schizochytrium* sp. [13, 14]. High oxygen supply shortens fermentation period and increases dry cell weight (DCW). Lipids, particularly polyunsaturated fatty acids (PUFAs), are highly susceptible to free radical attack. Acyl-CoA oxidase catalyzes the first step of β-oxidation of fatty acyl-CoA and generates hydrogen peroxide [15], and hydroxyl radical (·OH) and oxygen induce oxidation of unsaturated fatty acids and generate lipid peroxide [16]. Autolysis of resulting lipid peroxide generates a variety of lipid-derived aldehydes and ketones [17, 18]. Reactive oxygen species (ROS) and reactive aldehydes produced in this manner cause oxidation of proteins, lipids, and nucleic acids, with consequent disruption of DNA replication, loss of protein function, and even cell death [19, 20]. Microbial cells utilize various detoxification enzymes and non-enzymatic defensive mechanisms to protect cellular components from ROS and reactive aldehydes [21]. Major antioxidant enzymes include catalase (CAT), superoxide dismutase (SOD), and glutathione peroxidase (GPO). Reduced glutathione (GSH) and thioredoxin (TRX) are important non-enzymatic small molecules that scavenge ROS in cells [22, 23]. Glutathione reductase (GSR) catalyzes NADPH-dependent reduction of glutathione disulfide (GSSG) to GSH [23], and thioredoxin reductase (TRXR) utilizes NADPH for reduction of active-site disulfide of TRX [16]. Aldehyde dehydrogenase (ALDH) is an oxidizing enzyme involved in detoxification of both exogenous and endogenous aldehydes [24].

During microbial fermentation for production of lipids and PUFAs, generated ROS and reactive aldehydes impair cell metabolism and suppress lipid productivity. It is therefore crucial to control levels of intracellular oxidative species. In a study of the marine microalga *Crypthecodinium cohnii*, Liu et al. [25] reduced intracellular ROS concentration by adding the antioxidant sesamol, which significantly increased biomass and DHA content. Ren et al. [3], by adding the antioxidant ascorbic acid (9 g/L) to fermentation medium for *Schizochytrium* sp., obtained respective 16.16% and 30.44% increase in yields of DCW and DHA. Thus, reducing ROS levels of oleaginous microorganisms appears to be an effective strategy for enhancing cell robustness and DHA production. In studies of yeast, overexpression of ROS-scavenging enzymes reduced intracellular ROS, thereby promoting thermotolerance, robustness, and ethanol production [23, 26]. Xu et al. [16] produced an engineered *Yarrowia lipolytica* strain with high lipid titer (72.7 g/L) and high lipid content (81.4%) through combined overexpression of three enzymes (GSR, GPO, ALDH) of cellular oxidative stress defense pathways, and of glucose-6-phosphate dehydrogenase (ZWF), which generates NADPH for reduction of GSH and TRX disulfides.

In this study, we strengthened cellular oxidative stress defense pathways and aldehyde detoxification pathway in *Schizochytrium* sp. ATCC20888 through overexpression of several ROS-scavenging and aldehyde-scavenging enzymes, resulting in significant enhancement of cell growth, DHA production, and lipid production. In view of its robustness and high productivity, this engineered strain has strong potential as a *Schizochytrium* platform for efficient production of PUFAs and fatty acid-derived oleochemicals.

## Results and discussion

### Engineering oxidative stress defense pathways in *Schizochytrium* sp.

**Fig. 1:**
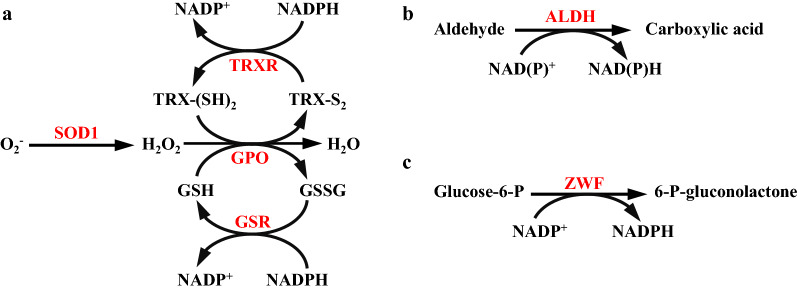
Genetic engineering of oxidative stress defense pathways to increase lipid and DHA production by *Schizochytrium*. SOD1: superoxide dismutase, GPO: glutathione peroxidase, GSR: glutathione disulfide reductase, TRXR: thioredoxin reductase, ZWF: glucose-6-phosphate dehydrogenase, ALDH: aldehyde dehydrogenase, GSH: glutathione, GSSG: glutathione disulfide, TRX-(SH)_2_: thioredoxin, TRX-S_2_: thioredoxin disulfide, Glucose-6-P: glucose-6-phosphate, 6-P-gluconolactone: 6-phosphate-gluconolactone

During aerobic fermentation, oxidation and peroxidation of lipids result in increased intracellular levels of ROS and reactive aldehydes, which affect cell growth and lipid accumulation [16]. We attempted to enhance the oxidative stress defense system of *Schizochytrium* to prevent or reduce oxidation of DHA and lipids. The key enzymes of the oxidative stress defense system for removal of oxygen free radicals and their products are GPO, GSR, SOD1, ALDH, and TRXR [22] (Fig. 1). GSR and TRXR both require NADPH as a reductant against oxidative stress. ZWF is an enzyme involved in the pentose phosphate pathway, the primary pathway for production of NADPH by cells [23] (Fig. 1). We therefore expected that overexpression of these antioxidant enzymes in *Schizochytrium* would help maintain redox homeostasis, and thereby promote cell growth and production of lipids and DHA.

The genes that encode GPO (*gpo*; YALI0_E02310g), GSR (*gsr*; YALI0_E18029g), SOD1 (*sod1*; YALI0_E12133g), and TRXR (*trxR*; YALI0_D27126g) were amplified from *Y. lipolytica* Po1f cDNA, and *aldH* (EG10036) was amplified from *E. coli* DH5α genomic DNA. BLAST search of the *Schizochytrium* sp. CCTCC M209059 genome [27] revealed one putative glucose-6-phosphate dehydrogenase-encoding gene (*zwf*; Additional file 2: Table S1). *zwf* was amplified from cDNA of *Schizochytrium* sp. ATCC20888. The above genes were cloned separately into pPICZαA under *ccg1* promoter or *TEF-1* promoter (Additional file 1: Figure S1), and corresponding transformants (Ogpo, Ogsr, Osod1, OtrxR, OaldH, Ozwf) were then obtained by electrotransformation.

### Overexpression of oxidative stress defense enzymes enhanced *Schizochytrium* sp. cell growth and lipid production

**Fig. 2:**
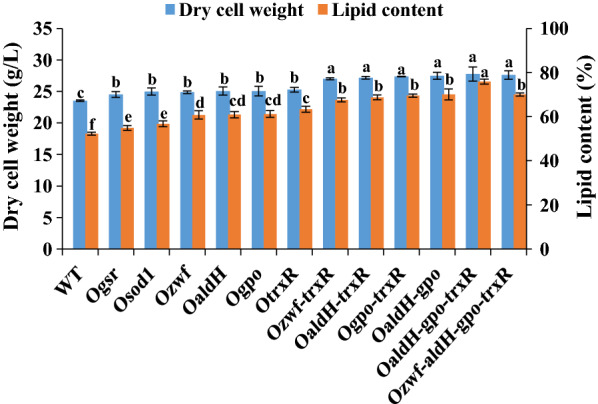
Effects on cell growth and lipid accumulation by overexpression of oxidative stress defense enzymes in various *Schizochytrium* strains as described in M&M. DCW (g/L) and lipid content (% DCW) are indicated on left and right Y-axes. Error bars: SD from three replicate experiments. Data were analyzed by one-way ANOVA and Duncan’s multiple range test, using SPSS V. 23.0. Differing lowercase letters indicate significant difference (p < 0.05) between values

Overexpression of antioxidant enzymes as above enhanced growth of *Schizochytrium* sp. ATCC20888 in shake-flask fermentation experiments (Fig. 2). DCW values for Ogsr, Osod1, Ozwf, OaldH, Ogpo, and OtrxR were increased 4.3–7.7% relative to WT value (23.5 g/L). Lipid production of overexpression strains was also increased significantly. Relative to WT value (52.3%), lipid content was much higher for OtrxR (63.3%), Ogpo (61.2%), OaldH (60.9%), and Ozwf (60.8%), and slightly higher for Ogsr (54.9%) and Osod1 (56.7%). Introduction of the control plasmid (pPICZ-ccg1p) had no effects on DCW, lipid accumulation, and DHA production of WT (Additional file 1: Figure S2). The results indicated that the increase of DCW, lipid production and DHA yields in the overexpression strains was due to overexpression of the oxidative stress defense genes. In yeast studies, enhancement of GSH and TRX antioxidant systems strongly promoted cell growth and production of ethanol and lipids [16, 23]. In the present study, enzyme overexpression had a stronger promoting effect on *Schizochytrium* cell growth and lipid production for TRXR than for other enzymes (Fig. 2). In mammalian cells, ALDH plays the major role in prevention of lipotoxicity caused by free radical attack on lipids [28]. OaldH had much higher DCW and lipid content than did WT, even though the *aldH* gene was from *E. coli*. ZWF catalyzed production of NADPH, which in addition to serving as reductant for GSR and TRXR also participates directly in fatty acid synthesis [29] – a process more conducive to lipid accumulation. Therefore, these findings demonstrate the essential roles of TRXR, ALDH, GPO, and ZWF in elimination of oxygen free radicals and reactive aldehydes in *Schizochytrium*.

**Fig. 3:**
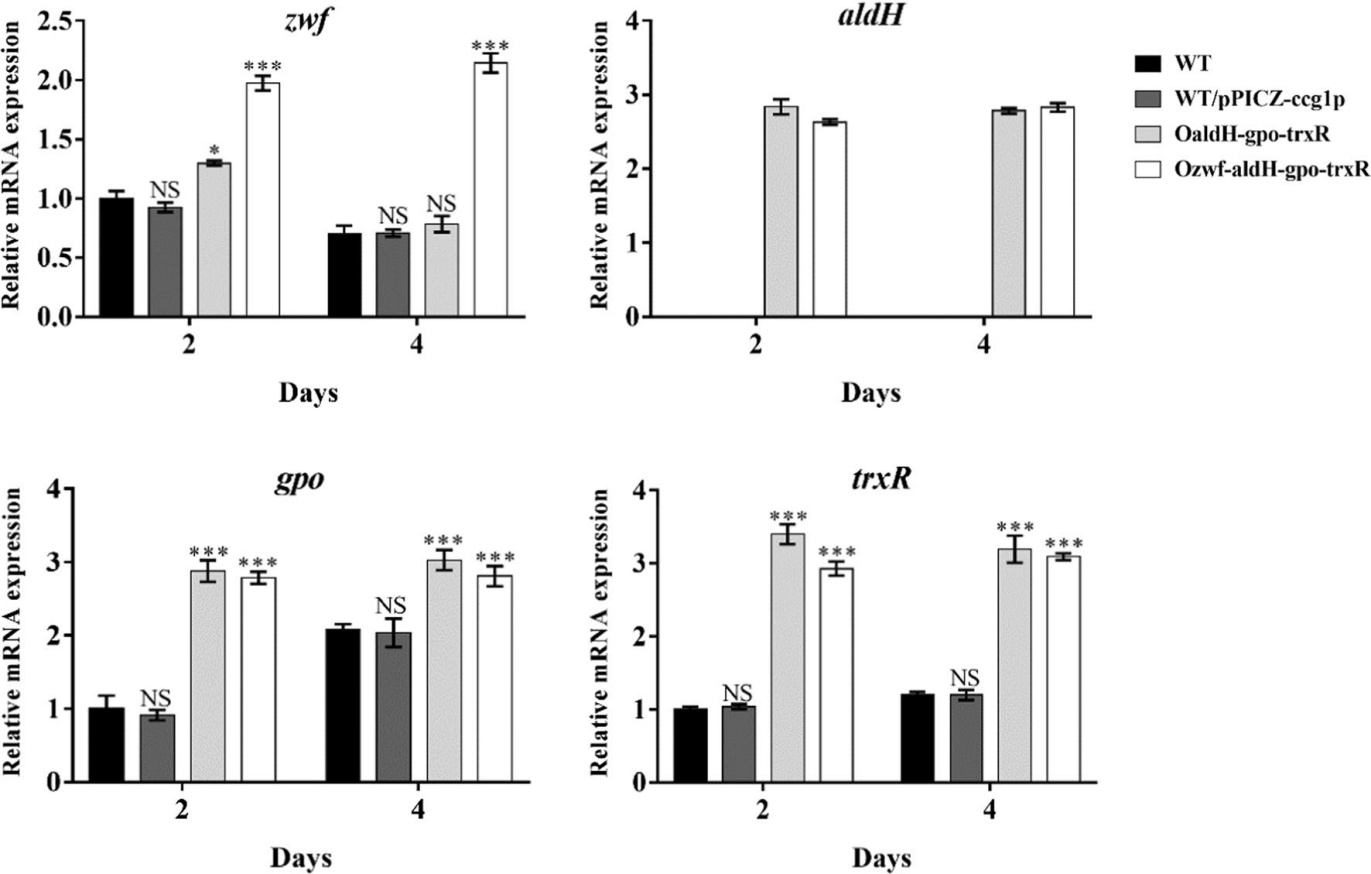
qRT-PCR analysis of the transcription levels of the *zwf*, *aldH*, *gpo*, and *trxR* genes in WT, WT/pPICZ-ccg1p, OaldH-gpo-trxR, and Ozwf-aldH-gpo-trxR. RNAs were isolated from cells grown in fermentation broth for 2 and 4 days. ***, p < 0.001; *, p < 0.05; NS, not significant by Student’s *t*-test

We next attempted to further increase lipid accumulation by overexpressing ZWF, ALDH, GPO, and TRXR in combinations of two, three, or four. Lipid yields of Ozwf-trxR, OaldH-trxR, Ogpo-trxR, and OaldH-gpo were notably higher than those of (respectively) Ozwf, OaldH, Ogpo, and OtrxR (Fig. 2; Table 1). That is, yields were increased by 48.8% in Ozwf-trxR, 52.0% in OaldH-trxR, 54.5% in Ogpo-trxR, and 56.9% in OaldH-gpo, relative to WT value (12.3 g/L) (Table 1). Antioxidant function of GSH and TRX in the yeast *Saccharomyces cerevisiae* was promoted by increased NADPH supply in previous studies [23, 26], and we achieved a similar result in *Schizochytrium* by coupling TRXR with ZWF (Fig. 2). Maximal DCW (27.8 g/L) and lipid yield (21.0 g/L) were achieved in OaldH-gpo-trxR (co-overexpression of ALDH, GPO, and TRXR). These values were respectively 18.3% and 70.7% higher than WT values (Table 1). However, overexpression of *zwf* in OaldH-gpo-trxR did not further improve DCW (27.6 g/L) and lipid yield (19.3 g/L) (Table 1; Additional file 1: Figure S2), and the possible reason might be that the simultaneous overexpression of the four genes increased the metabolic burden of *Schizochytrium* sp. RT-qPCR was performed to determine the transcription levels of *zwf*, *aldH*, *gpo*, and *trxR* genes in WT, WT/pPICZ-ccg1p, OaldH-gpo-trxR, and Ozwf-aldH-gpo-trxR. No significant difference in the transcription levels of the tested genes was observed between WT and the control strain WT/pPICZ-ccg1p, while the transcription levels of *aldH*, *gpo*, and *trxR* were greatly increased in OaldH-gpo-trxR, and the expression of the four tested genes was increased in Ozwf-aldH-gpo-trxR compared with WT (Fig. 3). In summary, coupling of oxidative stress defense pathways and aldehyde detoxification pathway in *Schizochytrium* notably promoted cell growth and reduced lipid oxidation.

**Table 1:** Fermentation characteristics of overexpression strains of *Schizochytrium* sp.

Strain	DCW (g/L)	Lipid yield (g/L)	Lipid content (%)	DHA yield (g/L)
WT	23.5 ± 0.1^b^	12.3 ± 0.1^d^	52.3^c^	4.3 ± 0.1^d^
Ozwf-trxR	27.0 ± 0.2^a^	18.3 ± 0.4^c^	67.6^b^	7.0 ± 0.1^c^
OaldH-trxR Ogpo-trxR OaldH-gpo OaldH-gpo-trxR Ozwf-aldH-gpo-trxR	27.2 ± 0.1^a^ 27.4 ± 0.2^a^ 27.5 ± 0.6^a^ 27.8 ± 1.1^a^ 27.6 ± 0.6^a^	18.7 ± 0.2^bc^ 19.0 ± 0.3^bc^ 19.3 ± 0.8^b^ 21.0 ± 0.4^a^ 19.3 ± 0.8^a^	68.7^b^ 69.5^b^ 70.1^b^ 75.8^a^ 70.0^b^	7.1 ± 0.1^bc^ 7.4 ± 0.2^b^ 7.3 ± 0.1^bc^ 8.8 ± 0.3^a^ 8.4 ± 0.2^a^

Data were analyzed by one-way ANOVA and Duncan’s multiple range test, using SPSS V. 23.0. Differing lowercase letters indicate significant difference (p < 0.05) between values

### Overexpression of ALDH, GPO, TRXR, and ZWF increased DHA production

**Fig. 4:**
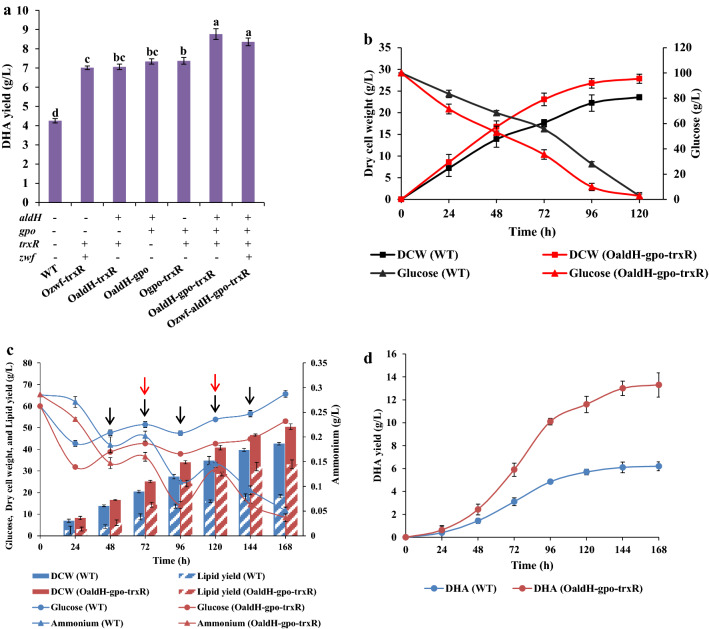
Effects of ALDH, GPO, TRXR, and ZWF overexpression on DHA production, cell growth, and carbon and nitrogen sources utilization. **a** DHA yield (g/L). Cells were cultured in fermentation medium for 120 h. **b** Growth curves and glucose consumption by WT and OaldH-gpo-trxR. **c**, **d** WT and OaldH-gpo-trxR strains were cultured for 168 h in shake flask fed-batch fermentation. The fermentation broth was collected every 24 h to determine the concentrations of glucose and ammonium, DCW, lipid and DHA yields. Black arrow: 25 g/L glucose was supplied to the culture every 24 h from 48 to 144 h. Red arrow: 2 g/L yeast extract was supplied to the fermentation culture at 72 h and 120 h, respectively

To investigate the possibility of increasing DHA production by enhancing oxidative stress defense pathways, we used gas chromatography to measure DHA yields of WT, Ozwf-trxR, OaldH-trxR, Ogpo-trxR, OaldH-gpo, OaldH-gpo-trxR, and Ozwf-aldH-gpo-trxR. DHA production in the overexpression strains was significantly increased (Fig. 4a), similarly to results for lipid production. DHA yields of Ozwf-trxR (7.0 g/L), OaldH-trxR (7.1 g/L), OaldH-gpo (7.3 g/L), and Ogpo-trxR (7.4 g/L) were 62.8-72.1% higher than that of WT (4.3 g/L). The highest DHA yield observed was that of OaldH-gpo-trxR (8.8 g/L) -- 104.7% higher than WT value. The DHA yield of Ozwf-aldH-gpo-trxR (8.4 g/L) was slightly lower than that of OaldH-gpo-trxR (Fig. 4a; Table 1). Therefore, OaldH-gpo-trxR was used for further analysis. The growth curves and glucose consumption curves of WT and OaldH-gpo-trxR showed striking differences. OaldH-gpo-trxR had faster growth rate, much faster glucose consumption rate, and higher DCW than WT (Fig. 4b). These findings demonstrate that overexpression of ALDH, GPO, and TRXR promoted robustness, carbon source uptake, and growth of *Schizochytrium*. Next, we performed fed-batch fermentation to test the ability of OaldH-gpo-trxR to produce lipids and DHA, together with the control strain. The initial concentrations of glucose and yeast extract were 60 g/L and 3 g/L, and then 125 g glucose and 4 g yeast extract were added in 5 and 2 times after 48 h and 72 h, respectively. The carbon and nitrogen sources consumption rates of OaldH-gpo-trxR were still much faster than those of WT (Fig. 4c). In both strains, the output of DCW, lipid and DHA increased rapidly from 48 to 96 h, and the synthesis of lipid and DHA became stable after 120 h (Fig. 4c, d). The DCW and the yields of lipid and DHA in OaldH-gpo-trxR reached 50.5, 33.1, and 13.3 g/L after 168 h of fermentation with 185 g/L glucose, which were increased by 18.5%, 80.9%, and 114.5% compared with WT (42.6, 18.3, and 6.2 g/L).

Various strategies have been used to increase DHA production of *Schizochytrium*, including traditional fermentation conditions optimization and metabolic engineering (Table 2). Chen et al. [30] increased DHA production of *Schizochytrium* sp. S056 through optimization of sea salt in the culture medium. Sun et al. [31] and Guo et al. [32] used adaptive laboratory evolution method and continuous feeding strategy to achieve DHA production of 38.1 g/L and 38.4 g/L in *Schizochytrium* sp. HX-308, respectively. The addition of inositol in the medium increased the lipid and DHA production of *Schizochytrium* sp. SR21 [33]. Recent studies also indicated that metabolic engineering is an efficient strategy to enhance lipid and DHA production of *Schizochytrium*. Wang et al. [12] increased lipid content and the proportion of odd fatty acids in *Schizochytrium* sp. S31 through increasing the intracellular NADPH supply and the acetyl-CoA carboxylase activity. Li et al. [11] overexpressed the malonyl CoA: ACP transacylase in *Schizochytrium* sp. MYA1381 and increased the DHA yield to 7.0 g/L in shake-flask fermentation and to 47.4 g/L in fed-batch fermentation. Here, we increased the DHA yield to 13.3 g/L in shake flask fed-batch fermentation through enhancing the oxidative stress defense pathways in *Schizochytrium* sp. ATCC20888 (Table 2). To our best knowledge, the DHA yield obtained by OaldH-gpo-trxR is at a high level in the shake-flask fermentation of *Schizochytrium* sp.

**Table 2:** DHA yield in *Schizochytrium* sp. from the literatures and this study

*Schizochytrium* sp.	Strategy	Cultivation mode	Yield (g/L)	Content (%TFA)	Reference
S056 HX-308	Optimization of sea salt ALE	Shake flask 1,500-L fermentor	8.0 38.1	44.4 53.3	[30] [31]
HX-308	Addition of ascorbic acid	5-L bioreactor	38.3	54.5	[3]
SR21	Addition of inositol	Shake flask	8.5	37.3	[33]
HX-308 MYA1381 ATCC20888 ATCC20888 ATCC20888	Continuous feeding Metabolic engineering Metabolic engineering Metabolic engineering Metabolic engineering	Fed-batch Shake-flask/ Fed-batch Shake-flask Shake-flask Shake-flask/ Fed-batch	38.4 7.0/ 47.4 3.5 6.4 8.8/ 13.3	55.0 42.9 N.C 37.9 42.8	[32] [11] [12] [10] This study

ALE: Adaptive laboratory evolution, N.C.: not calculated

**Fig. 5:**
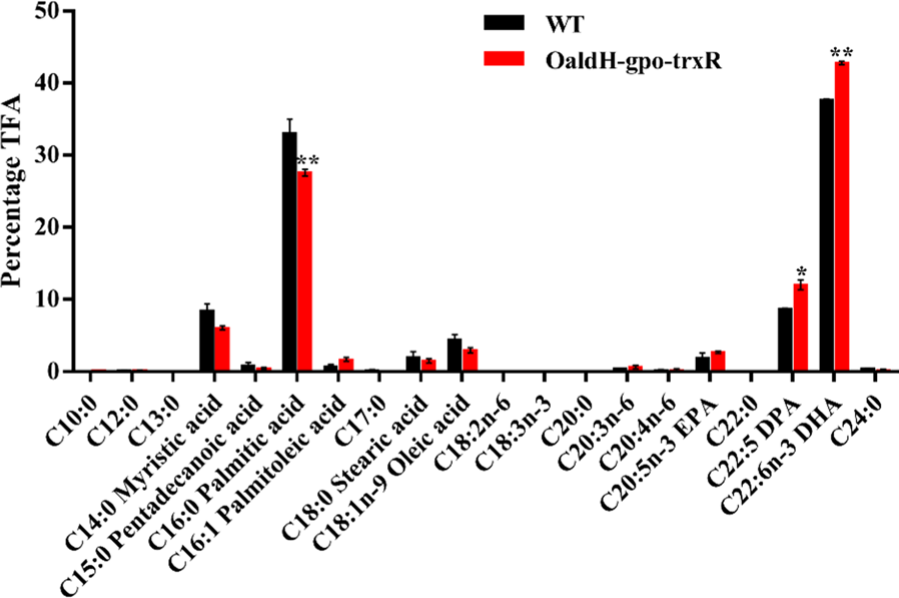
Fatty acid composition (TFA, %) of WT and OaldH-gpo-trxR. Cells were cultured in fermentation medium for 120 h. *, p < 0.05; **, p < 0.01 (Student’s *t*-test)

Overexpression of ALDH, GPO, and TRXR affected fatty acid composition as well as increasing DHA yield. Fatty acid composition analysis revealed that, relative to WT values, the percentage of TFAs corresponding to major saturated fatty acids (myristic acid [C14:0] and palmitic acid [C16:0]) in OaldH-gpo-trxR was reduced, percentage for docosapentaenoic acid (DPA, C22:5) was slightly increased, and percentage for DHA was significantly increased (42.8%; WT value was 37.7%) (Fig. 5). In a previous study, PUFAs were readily oxidized for protection of cells from oxidative damage. Addition of omega-3 fatty acids to human cells reduced intracellular ROS formation, suggesting that these molecules may function as antioxidants [34]. During aerobic fermentation process in our study, PUFAs (DHA and DPA) in TFAs of *Schizochytrium* were more readily oxidized than were saturated fatty acids for protection of cells from ROS damage, and maintenance of intracellular redox homeostasis. DHA production in both *Schizochytrium* sp. and *Crypthecodinium cohnii* [3, 25] was enhanced by addition of antioxidants. We also observed that enhancement of oxidative stress defense pathways in *Schizochytrium* increased production of PUFAs (particularly DHA).

### Overexpression of ALDH, GPO, and TRXR reduced intracellular ROS levels

**Fig. 6:**
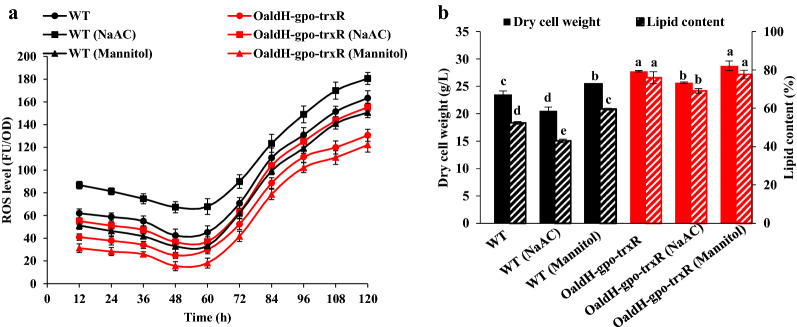
Effects of ALDH, GPO, and TRXR overexpression on ROS levels in WT and OaldH-gpo-trxR. **a** ROS level (FU/OD_600_). **b** DCW (g/L) and lipid content (% DCW). Cells were cultured for 120 h in fermentation medium with or without 0.3 M NaOAc or 60 mM mannitol

To examine the possibility that enhanced oxidative stress defense ability was the cause of increased growth rate and lipid accumulation in our overexpression strains, we measured intracellular ROS levels in WT and OaldH-gpo-trxR during fermentation process. In both strains, ROS levels declined gradually at the beginning of fermentation, reached minimal values at 48 h, and subsequently showed rapid increase (Fig. 6a); that is, intracellular ROS increased rapidly during lipid synthesis/ accumulation stage. OaldH-gpo-trxR, relative to WT, showed much lower ROS level but much higher DCW and lipid yield (Fig. 6b). In previous studies, weak acids, *e.g.*, acetic acid, were found to promote ROS accumulation and induce oxidative stress [35, 36]. Mannitol scavenged hydroxyl radicals and protected cells from oxidative stress [37]. We therefore added 0.3 M sodium acetate (NaOAc) or 60 mM mannitol to fermentation medium to evaluate the ROS-scavenging ability of OaldH-gpo-trxR. In WT, NaOAc addition resulted in increase of ROS level from 163.3 to 180.6 FU/OD_600_ at 120 h, and reductions of DCW and lipid yield by 12.8% and 28.5%, respectively (Fig. 6). In OaldH-gpo-trxR, NaOAc addition also increased ROS level and reduced DCW and lipid content, indicating that cell growth and lipid accumulation were inhibited by increased ROS. In NaOAc-treated OaldH-gpo-trxR, relative to untreated WT, ROS level was lower (Fig. 6a), and DCW and lipid content were higher (Fig. 6b). Addition of mannitol resulted in reduced ROS level and increased DCW and lipid content for both WT and OaldH-gpo-trxR. ROS level of mannitol-treated WT was higher than that of untreated OaldH-gpo-trxR (Fig. 6a). These findings, taken together, indicate that ROS-scavenging ability of *Schizochytrium* was notably increased by overexpression of ZWF, ALDH, GPO, and TRXR. Evidently, enhancement of oxidative stress defense pathways and aldehyde detoxification pathway resulted in detoxification of intracellular ROS and lipid-derived aldehydes, prevented reactive radicals from attacking nucleophilic centers of bioactive molecules, and thereby improved cell robustness and lipid production.

## Conclusions

We overexpressed six ROS-scavenging and aldehyde-scavenging enzymes in *Schizochytrium* sp. for the purpose of improving cell robustness and DHA production. Overexpression of ZWF, ALDH, GPO, and TRXR strongly promoted cell growth, lipid yield, and DHA production. We generated a DHA high-producer, OaldH-gpo-trxR, that attained 50.5 g/L DCW, 33.1 g/L lipid yield, and 13.3 g/L DHA production in shake flask fed-batch fermentation, respectively, 18.5, 80.9, and 114.5% increases relative to WT values. Our findings illustrate a new method for ROS reduction in *Schizochytrium* sp., and provide an effective strategy for generation of DHA high-producing strains.

## Methods

### Microorganisms and culture conditions

*Schizochytrium* sp. ATCC20888, used as wild-type strain (WT), was preserved in 20% (v/v) glycerol at -80°C. Strains used in the study are listed in Table 3. *E. coli* was cultured in LB broth or agar, added when necessary with 50 μg/mL zeocin. *Schizochytrium* sp. were cultured under conditions and media as in our previous study [10]. All fermentation experiments were performed in triplicate, and results from a representative batch are presented. For ROS analysis, cells were cultured for 120 h in fermentation medium supplemented with 0.3 M sodium acetate or 60 mM mannitol, on a rotary shaker (250 rpm).

**Table 3:** Strains used in this study

Strain	Description	Source or reference
*Schizochytrium* sp.		
ATCC 20888	wild-type strain (WT)	American Type Culture Collection
WT/pPICZ-ccg1p	WT carrying control plasmid pPICZ-ccg1p	This study
Ogsr	*gsr* overexpression strain	This study
Osod1	*sod1* overexpression strain	This study
Ozwf	*zwf* overexpression strain	This study
OaldH	*aldH* overexpression strain	This study
Ogpo	*gpo* overexpression strain	This study
OtrxR	*trxR* overexpression strain	This study
Ozwf-trxR	*zwf* and *trxR* co-overexpression strain	This study
OaldH-trxR	*aldH* and *trxR* co-overexpression strain	This study
Ogpo-trxR	*gpo* and *trxR* co-overexpression strain	This study
OaldH-gpo	*aldH* and *gpo* co-overexpression strain	This study
OaldH-gpo-trxR	*aldH*, *gpo*, and *trxR* co-overexpression strain	This study
Ozwf-aldH-gpo-trxR	*zwf*, *aldH*, *gpo*, and *trxR* co-overexpression strain	This study
*E. coli*		
JM109	General cloning host for plasmid manipulation	Laboratory stock

The fed-batch fermentation was carried out in the shake flasks and started with an initial glucose level of 60 g/L and a yeast extract concentration of 3 g/L. The carbon source supplementation strategy is to supply 25 g/L glucose to the culture every 24 h from 48 to 144 h. The nitrogen source supplementation strategy is to supply 2 g/L yeast extract to the fermentation culture at 72 h and 120 h, respectively. The fermentation broth was collected every 24 h to determine the concentrations of glucose and ammonium, DCW, lipid and DHA yields.

### Plasmid construction

For construction of oxidative stress defense pathways-related plasmids, *aldH* was amplified from *E. coli* DH5α genomic DNA, *gpo*, *gsr*, *sod1*, and *trxR* were amplified from *Y. lipolytica* Po1f cDNA, and *zwf* was amplified from *Schizochytrium* sp. ATCC20888 cDNA. *aldH*, *trxR*, and *zwf* were promoted by *ccg1* promoter and terminator amplified from *Neurospora* expression vector pCCG.N-3xMyc [38], and *gpo*, *gsr*, and *sod1*were promoted by *TEF-1* promoter and *CYC-1* terminator amplified from yeast expression vector pPICZαA [39]. Primer pairs used in the study are listed in Additional file 2: Table S2. *Eco*RI/*Kpn*I-digested promoter, *Kpn*I*/Not*I-digested gene, and *Not*I/*Xba*I-digested terminator were ligated simultaneously into *Eco*RI/*Xba*I-digested pPICZαA to generate single gene overexpression plasmid (Additional file 1: Figure S1). Ligation reactions were performed overnight at 16°C using T4 DNA Ligase (TaKaRa Bio; Shiga, Japan). For overexpression of two, three, or four genes, promoter-gene-terminator expression cassette was amplified from single gene overexpression plasmid and inserted into *Xba*I-digested corresponding overexpression plasmid using Seamless Cloning and Assembly Kit (Clone Smarter Technologies; Houston, TX, USA). pPICZαA-ccg1p-lacZ [10] was digested by *Kpn*I*/Not*I to produce the control plasmid pPICZ-ccg1p.

### Strain construction

*Pme*I-linearized overexpression plasmids were transformed into *Schizochytrium* competent cells to generate corresponding overexpression strains. Transformation of *Schizochytrium* was performed as described previously [40] with some modifications. In brief, *Schizochytrium* cells were cultured in seed medium for 24 h, and were harvested by centrifugation (5900 *g*, 4°C, 5 min) (HITACHI; Tokyo, Japan), washed with ice-cold sterile water and 1 M sorbitol, and resuspended in 1 M sorbitol. Plasmids were linearized by *Pme*I before electroporation. Linearized plasmid DNA (5–10 μg) and competent cells were placed in a 0.1-cm-gap cuvette for electroporation (1.5 kV, 200 Ω, 50 μF, twice). Cells were then added with 1 mL seed medium, incubated 4 h at 28°C, spread on glucose/ peptone/ yeast extract (GPY) plates with 40 μg/mL zeocin, and grown at 28°C for selection of transformants.

### Analytical methods

For determination of DCW, pellets from 40 mL fermentation broth were freeze-dried (24–48 h) to constant weight. Glucose concentration was determined by 3,5-dinitrosalicylic acid (DNS) method [41]. Total lipid content and fatty acid composition were analyzed as described previously [42, 43]. For lipid extraction, ~ 0.3 g freeze-dried pellet was mixed with 6 mL of 4 M HCl for 30 min, incubated in boiling water for 8 min, added with 16 mL methanol/chloroform (1:1, v/v), mixed vigorously, and centrifuged. The lower phase was transferred to a tube and evaporated under gentle nitrogen stream. Fatty acid methyl esters (FAMEs) were prepared from 30 mg lipid sample and analyzed by gas chromatography (model GC522, Shanghai Wufeng Scientific Instruments Co.) with J&W DB23 capillary column (30 m × 0.25 mm i.d.) (Agilent Technologies; Santa Clara, CA, USA), using nitrogen as carrier gas, injector temperature 250°C, and injection volume l μL. Column temperature was raised from 150 to 200°C at 5°C per min, kept at 200°C for 1 min, further raised to 230°C at 4°C per min, and kept at 230°C for 9 min.

### Quantitative real-time PCR (qRT-PCR) analysis

Total RNAs were extracted from *Schizochytrium* sp. cells using TRIzol reagent (Tiangen; China) according to the manufacturer’s protocol. The cDNA was prepared with M-MLV (RNase H^−^; TaKaRa Bio; Shiga, Japan) using oligo-dT18 from 4 µg of total RNAs. qRT-PCR was performed as described previously [10]. The primers used for qRT-PCR are listed in Additional file 2: Table S2. The relative expression levels were determined according to the comparative Ct method, using actin as the internal control. Since *aldH*, *gpo* and *trxR* were from *E. coli* and *Y. lipolytica,* the transcriptional levels of the endogenous genes were analyzed in WT and WT/pPICZ-ccg1p (*Schizochytrium* sp. does not contain the *aldH* gene), and of the transformed genes were analyzed in OaldH-gpo-trxR and Ozwf-aldH-gpo-trxR.

### Determination of intracellular ROS levels

These levels were determined using an ROS assay kit (Beijing Solarbio Science & Technology Co.; China) as per manufacturer’s instructions. *Schizochytrium* cells were suspended in 10 μM DCFH-DA, incubated for 20 min at 37°C with occasional gentle stirring, and washed 3× with PBS to remove unincorporated DCFH-DA. Fluorescence intensity was measured using a multifunctional plate reader (Molecular Devices; San Jose, CA, USA) with excitation and emission wavelengths 488 nm and 525 nm, respectively.

## Supplementary Information

**Additional file 1**: **Figure S1**. Schematic illustration of genetic constructs used for genomic integration. **Figure S2**. Effects of ZWF, ALDH, GPO, and TRXR overexpression on cell growth, lipid accumulation and DHA production. **a** DCW (g/L). **b** Lipid content (% DCW). **c** DHA yield (g/L). Cells were cultured in fermentation medium for 1 to 5 days.

**Additional file 2**: **Table S1**. The open reading frame of *zwf* gene in *Schizochytrium* sp. **Table S2**. Primers used in this study.

## Data Availability

All data supporting the conclusions of this article are included in the manuscript and in the additional information.

## Abbreviations

ALDH: Aldehyde dehydrogenase
CAT: Catalase
DCW: Dry cell weight
DHA: Docosahexaenoic acid
DNS: 3,5-Dinitrosalicylic acid
DPA: Docosapentaenoic acid
FAMEs: Fatty acid methyl esters
GPO: Glutathione peroxidase
GSH: Glutathione
GSR: Glutathione reductase
GSSG: Glutathione disulfide
NaOAc: Sodium acetate
PBS: Phosphate buffer saline
PUFAs: Polyunsaturated fatty acids
ROS: Reactive oxygen species
SOD1: Superoxide dismutase
TFAs: Total fatty acids
TRX: Thioredoxin
TRXR: Thioredoxin reductase
WT: Wild type
ZWF: Glucose-6-phosphate dehydrogenase
