# Supplementary material for: Overproduction of docosahexaenoic acid in Schizochytrium sp. through genetic engineering of oxidative stress defense pathways
Source: Biotechnol Biofuels. 2021 Mar 16;14:70. doi: 10.1186/s13068-021-01918-w (PMC7968238; doi:10.1186/s13068-021-01918-w)
Supplement: Supplementary file 1 — Additional file 1: Figure S1. Schematic illustration of genetic constructs used for genomic integration. Figure S2. Effects of ZWF, ALDH, GPO, and TRXR overexpression on cell growth, lipid accumulation and DHA production. a DCW (g/L). b Lipid content (% DCW). c DHA yield (g/L). Cells were cultured in fermentation medium for 1 to 5 days. [file 13068_2021_1918_MOESM1_ESM.pptx]

## Slide 1
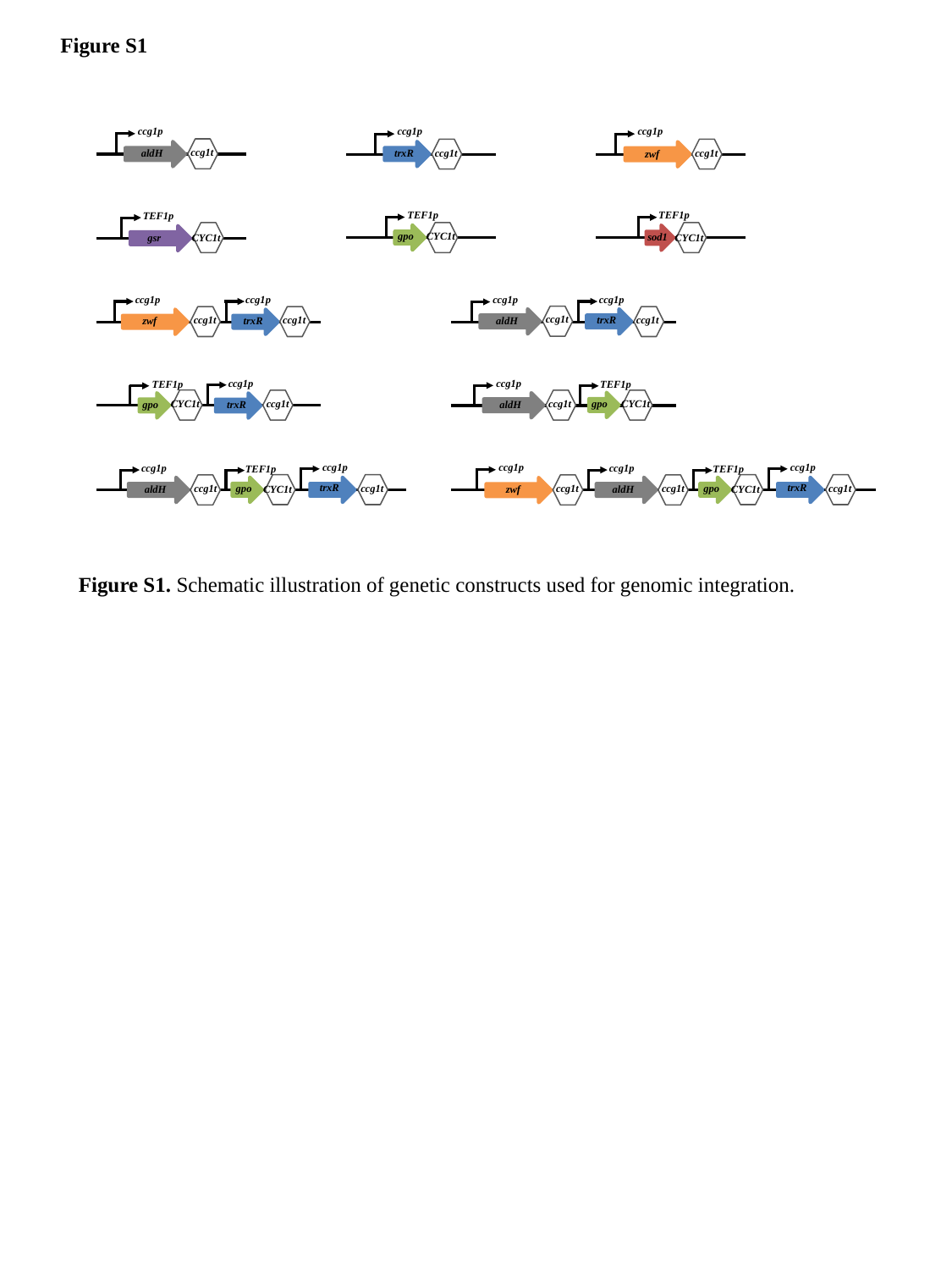

Figure S1
ccg1p
ccg1t
aldH
ccg1p
ccg1t
trxR
ccg1p
ccg1t
zwf
TEF1p
gpo
CYC1t
TEF1p
sod1
CYC1t
TEF1p
gsr
CYC1t
ccg1p
ccg1p
ccg1t
ccg1t
zwf
trxR
ccg1p
ccg1p
ccg1t
ccg1t
trxR
aldH
ccg1p
TEF1p
ccg1t
CYC1t
gpo
trxR
ccg1p
TEF1p
ccg1t
gpo
CYC1t
aldH
ccg1p
ccg1p
TEF1p
trxR
ccg1t
ccg1t
gpo
CYC1t
aldH
ccg1p
ccg1p
TEF1p
trxR
ccg1t
ccg1t
gpo
CYC1t
aldH
ccg1p
ccg1t
zwf
Figure S1. Schematic illustration of genetic constructs used for genomic integration.

## Slide 2
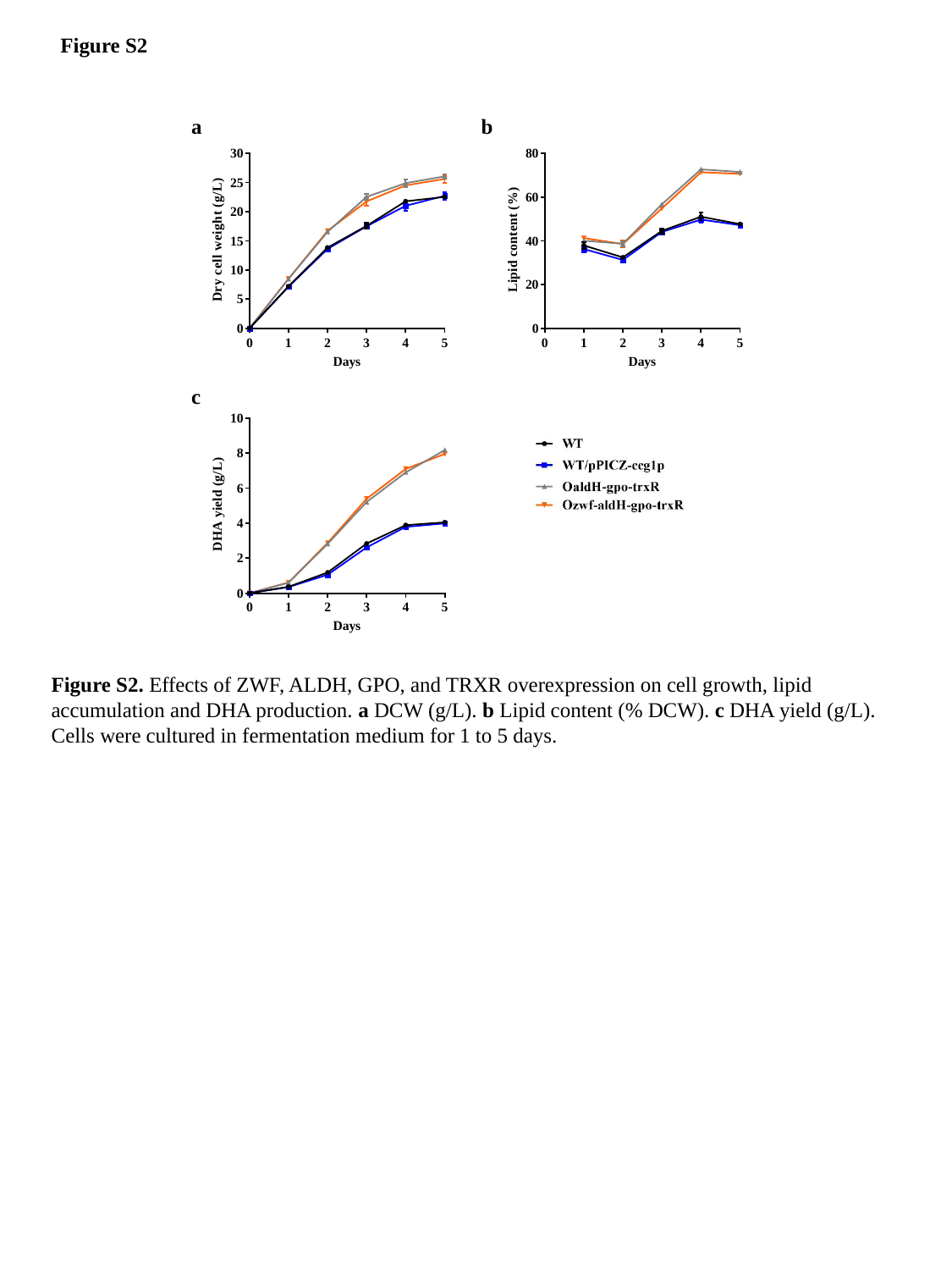

Figure S2
a
b
c
Figure S2. Effects of ZWF, ALDH, GPO, and TRXR overexpression on cell growth, lipid accumulation and DHA production. a DCW (g/L). b Lipid content (% DCW). c DHA yield (g/L). Cells were cultured in fermentation medium for 1 to 5 days.
