# Supplementary material for: Overproduction of docosahexaenoic acid in Schizochytrium sp. through genetic engineering of oxidative stress defense pathways
Source: Biotechnol Biofuels. 2021 Mar 16;14:70. doi: 10.1186/s13068-021-01918-w (PMC7968238; doi:10.1186/s13068-021-01918-w)
Supplement: Supplementary file 2 — Additional file 2: Table S1. The open reading frame of zwf gene in Schizochytrium sp. Table S2. Primers used in this study. [file 13068_2021_1918_MOESM2_ESM.docx]

**Table S1 The open reading frame of *zwf* gene in *Schizochytrium* sp.**

| **Name** | **Sequence (5’-3’)** |
| --- | --- |
| *zwf* | ATGTTGGACCAAATGATGAGCGAGGAGGAGTACGCTCCCGCAGAGTTCCT  CGACCCGGACCAGGAGACTTGGCATGAGGAGAGGCTGACCTTGGTCGTCT  TTGGAGCCTCGGGAGACCTGGCCAAGAAGAAGACGTTCCCGGCCATCTTT  GACCTCTTTCGCGTCGGCGCCCTCCCGCGACTGACCATTGTGTGCGGCTA  TGCAAGGTCCAAGATGAGCGACGAGGATTTTCGCTCGCGTATCCGTGAAT  ACCTCGAGGGAAAGGGCTCCGAGGAGGAGCTCGAGCGCTTCCTCGACATT  TGCATCTACCGCAGCGGACAGTACGACGGCGTCGAGGAATTCAAGGAGAC  AAGCGCCGAGCTCCGCGCCCTCCACGAGTGCAAGGACGACCAGGAGGTAG  AGAATCGCATGTACTACCTGGCCATCCCTCCCAACCAGTTTCTCAACACG  ATCAAGACTACTCGCGAGGGAGGTATGTCCAAAACCGGATGGACTCGCGT  CGTTGTGGAGAAGCCCTTTGGCCAAGACACCGAATCGGCGAAAAAGCTTG  GCGACGACATTAGCCAGTATTTTGACGAGTCCCACCTGTACCGCATCGAT  CACTACCTGGGCAAGGAGATGGTGCAGAACCTCGTCAGTCTTCGCTTCGG  CAACGCCTTCCTTGAGCCTCTATTTAATCGTGACCACGTCAAATCCGTCA  TTATCTCCTTCAAGGAGCCCTTTGGCACAGAGGGCCGCGGCGGCTACTTT  GACTCGTATGGAATTATCCGCGACGTCATGCAAAACCATCTTATGCAGCT  TCTCAGCATCATCGCAATGGAGCCGCCCGTAAAGGTTGCCGGCACCACCA  AAGACGGAACCGACTATTCGCGCTTTGTTCGCGATGAAAAGGTCAAGGTC  CTTGCTGCTGTCAAACCCTGGTCCCTCGATGACGTCGTTCTCGGCCAGTA  CGTCTCCAACGGGGACAAGCCGGGCTACCTCGACGATGACACCGTCCCCG  AGGGTTCCAACCAGCCCACCTACGCGGCTGTGCGCATGTTCATTCATAAC  AAGCGCTGGGATGGCGTGCCTTTTATCATGAAGGCTGGCAAGGCGCTCGA  CGAGAAAAAGTGCGAGGTGCGTTTCCAGTTCCGTGACCCGGCCGGCGCAG  CCGCCATGTTCGGCGAGTCGCGCATTCCGCGTAACGAGCTTGTACTCCGC  CTCCAGCCCCAGGAGAGCATCTACATGAAGCTCAACGTGAAGAAGCCCGG  TCTCGCCACTACCATGGTGCAGAGTTCGCTCGACCTCGACTACACAGATC  GCTACGACGATCACCAGATTCCGGAGGCCTACACGCGTCTTTTGCTCGAC  GTTTTGCGTGGAAAGCAAGCAACCTTTGTTCGCGACGACGAGCTTCTTGC  TGCCTGGGAAATTGTCACGCCTCTTTTGGAGGAAATCGAGTCGGGTGATG  TCAAGCCCATTCCGTACGAGTACGGATCGCGTGGACCTGCCGAGGCTGAC  GAGCTCGTTAAGGACTCTGGATACGTTCGCAACGCCGAGTACGCAGCACG  CTACAACGACTGGAAGGAGTCCAAGAAGAAGTAA |

**Table S2 Primers used in this study**

| **Purpose** | **Primer** | **DNA sequence (5'–3')** | **Length (bp)** |
| --- | --- | --- | --- |
| For genes overexpression | ccg1p-Fw  ccg1p-Rev  ccg1t-Fw  ccg1t-Rev  aldH-Fw | CCGGAATTCTCGACGGTATCGATAAGC (*Eco*RI)  GGGGTACCTTTGGTTGATGTGAGGGG (*Kpn*I)  ATAAGAATGCGGCCGCCTCCATGGCCTCCTCGAC (*Not*I)  GCTCTAGACGGCTGCAACAAGATTGT (*Xba*I)  GGGGTACCATGAATTTTCATCATCTG (*Kpn*I) | 869  473  1488  960  1584  411  318  1413  507  465  2302  2302  1236 |
|  | aldH-Rev | ATAAGAATGCGGCCGCTCAGGCCTCCAGGCTTAT (*Not*I) |  |
|  | trxR-Fw | GGGGTACCATGACCCACAGCCCAGTT (*Kpn*I) |  |
|  | trxR-Rev | ATAAGAATGCGGCCGCCTATTCCTCCTCAGCAAG (*Not*I) |  |
|  | zwf-Fw | GGGGTACCATGTTGGACCAAATGATG (*Kpn*I) |  |
|  | zwf-Rev  TEF1p-Fw  TEF1p-Rev  CYC1t-Fw  CYC1t-Rev | ATAAGAATGCGGCCGCTTACTTCTTCTTGGACTC (*Not*I)  CCGGAATTCTTTTCTCTTTCAGTGACC (*Eco*RI)  GGGGTACCCCAGGCCAGGGTGTTGTC (*Kpn*I)  ATAAGAATGCGGCCGCTCATGTAATTAGTTATGT (*Not*I)  GCTCTAGAGCAAATTAAAGCCTTCGA (*Xba*I) |  |
|  | gsr-Fw | GGGGTACCATGGCTTCTATCCCCCAT (*Kpn*I) |  |
|  | gsr-Rev | ATAAGAATGCGGCCGCCTATCTCATGGTCACCAG (*Not*I) |  |
|  | gpo-Fw | GGGGTACCATGTCCGCCGAGAAAACC (*Kpn*I) |  |
|  | gpo-Rev | ATAAGAATGCGGCCGCTTAGGGCTTTTTGAGGAG (*Not*I) |  |
|  | sod1-Fw | GGGGTACCATGGTCAAGGCTGTCGCT (*Kpn*I) |  |
|  | sod1-Rev | ATAAGAATGCGGCCGCTTAGGCGGTAAGACCAAT (*Not*I) |  |
|  | trxRS1-Fw | ACACAATCTTGTTGCAGCCGTCGACGGTATCGATAAGC |  |
|  | trxRS1-Rev  trxRS2-Fw  trxRS2- Rev | TTCTGAGATGAGTTTTTGTTTCTAGACGGCTGCAACAAGATTGT (*Xba*I)  GCTCGAAGGCTTTAATTTGCTCGACGGTATCGATAAGC  TTCTGAGATGAGTTTTTGTTTCTAGACGGCTGCAACAAGATTGT (*Xba*I) |  |
|  | gpoS-Fw | ACACAATCTTGTTGCAGCCGTTTTCTCTTTCAGTGACC |  |
|  | gpoS-Rev | TTCTGAGATGAGTTTTTGTTTCTAGAGCAAATTAAAGCCTTCGA (*Xba*I) |  |
| For verification of transformants | AOX-Fw | GACTGGTTCCAATTGACAAGC | 130  107  87  90  83  109  105 |
| For qRT-PCR | AOX-Rev  actin-QP-Fw  actin-QP-Rev  aldH-QP-Fw  aldH-QP-Rev  gpo-QPS-Fw  gpo-QPS-Rev  gpo-QPYl-Fw  gpo-QPYl-Rev  trxR-QPS-Fw  trxR-QPS-Rev  trxR-QPYl-Fw  trxR-QPYl-Rev  zwf-QP-Fw  zwf-QP-Rev | GCAAATGGCATTCTGACATCC  GCGACATCAAGGAGAAGC  GAAGGACGGCTGGAAGAG  GCTTTATTCGGGAAGGCG  ATTCGGGTCCACATCCAC  GCACTTGCCAAAGATGAC  ATCCTGATACCCGCTCTC  ACCAATACCGCTTTCTAC  GATGAGCACGACCTTGCC  GATGACGGCGAAGATACG  GCACCGATGATGACAACG  AGCAGTCCATCCGATTCG  GGCTCCTCGTCCTCATTG  CCGCATTCAGCCCAACAG  GGGTCGTCTCCGAGCAGG |  |

Underlining: Restriction endonuclease site
